# An Object Lesson for Antivaccinists

**Published:** 1896-06

**Authors:** 


### AN OBJECT LESSON FOR ANTIVACCINISTS.

APROPOS of the centennial of the great discovery made by Jenner in 1796, the case of the city of Gloucester, England, offers even at this late day a striking example from which to draw the lesson of the beneficial results to mankind of this discovery. The incident gains additional interest when we recall the fact that

Jenner was a native of the county of which Gloucester is the county-seat. Verily, "a prophet is not without honor save in his own *county*."

Gloucester, according to the *Journal of the American Medical Association*, May 9th, has for years been the center of an active anti-vaccination agitation. The authorities who were charged with the duty of securing vaccination have persistently and successfully refused to meet their obligations in this particular. The result of this neglect of duty is not hard to infer. Small-pox has been in practical possession of the town. During the first three and one-half months of the current year no less than 1,300 cases had been reported. The population of Gloucester is 42,000. In the period named the proportion of inhabitants attacked by the disease was one in every thirty-two or thirty-three. As has been the case in other epidemics of small-pox, the heaviest percentage of mortality has been among the unvaccinated, and where unvaccinated patients have not died they have been the greatest sufferers, in many cases becoming permanently disfigured or blind. Vaccinated infants have escaped, and where adults who had been vaccinated in infancy have been attacked the severity of the disease has been greatly lessened.

The *British Medical Journal*, April 25th, reports that the epidemic has promoted vaccination throughout England ; the guardians of the Gloucester Union even, the negligent officials referred to in the preceding paragraph, have been scared into doing their duty by enforcing the law in regard to vaccination. The result of this changed attitude on their part, aided by the vigorous action of the Town Council, has been such that within a few weeks no less than 25,000 vaccinations and re-vaccinations have been performed.

